# Effects and mechanisms of auricular electroacupuncture on gastric hypersensitivity in a rodent model of functional dyspepsia

**DOI:** 10.1371/journal.pone.0174568

**Published:** 2017-03-28

**Authors:** Jingzhu Zhou, Shiying Li, Yinping Wang, Yong Lei, Robert D. Foreman, Jieyun Yin, Jiande D. Z. Chen

**Affiliations:** 1 Veterans Research and Education Foundation, VA Medical Center, Oklahoma City, Oklahoma, United States of America; 2 Department of Physiology, University of Oklahoma Health Science Center, Oklahoma City, Oklahoma, United States of America; 3 Department of Acupuncture and Moxibustion, The First Affiliated Hospital with Nanjing Medical University, Nanjing, Jiangsu Province, China; 4 Ningbo Pace Translational Medical Research Center, Beilun, Ningbo, China; 5 Center of Neurogastroenterology, Johns Hopkins Medicine, Baltimore, Maryland, United States of America; University of California Los Angeles, UNITED STATES

## Abstract

**Background:**

Functional dyspepsia (FD) is a common functional gastrointestinal disease, and abdominal pain is one of the main symptoms. The aim of this study was to explore the effects and mechanisms of auricular electro-acupuncture (AEA) on gastric hypersensitivity in a rodent model of FD.

**Methods:**

Ten-day-old pups were gavaged with 0.2 ml of 0.1% iodoacetamide daily for 6 days. AEA at the “stomach” point with different parameters or sham-EA was performed on 8-week-old animals. Gastric sensitivity to gastric distention was measured under different conditions. Autonomic functions were assessed from the spectral analysis of heart rate variability (HRV) derived from the electrocardiogram. Naloxone was injected intraperitoneally before AEA to explore the opioid mechanism. Gastric emptying was measured at the end of the study.

**Results:**

1) Gastric sensitivity to gastric distention was higher in the FD rats. AEA with parameters of 0.1s on, 0.4s off, 100Hz, 0.3ms and 0.4–0.5mA, but not other parameters or sham-EA, decreased gastric hypersensitivity in the FD rats. Naloxone did not block the effect of AEA. 2) Lower vagal activity and higher sympathovagal ratio were noted in the FD rats, compared with the controls. AEA increased vagal activity and improved sympathovagal imbalance.

**Conclusions:**

AEA ameliorates gastric hypersensitivity in FD rats and this effect may be attributed to the improvement of sympathovagal balance.

## Introduction

Functional dyspepsia (FD) is one of functional gastrointestinal diseases with major complaints of postprandial fullness, early satiety, epigastric pain and burning sensation. The prevalence of FD ranges from 11.5%-29.2% worldwide [1]. Epigastric pain is one of dominating reasons for a FD patient to seek medical help. The cost of FD in 2009 was evaluated up to $18.4 billion in USA [2]. However, the treatment for FD is limited and unsatisfactory. Helicobacter pylori eradication is suggested if helicobacter pylori infection exists [3]. Proton pump inhibitors, prokinetics, antidepressants and serotonin reuptake inhibitors are used to relieve symptoms [4, 5].

Although the pathophysiology of FD is not fully understood, gastric hypersensitivity and abnormal gastric motility are commonly noticed in patients with FD. Gastric hypersensitivity has been reported to be associated with the symptoms of postprandial pain, belching, and weight loss in FD patients [6]. In addition, hypersensitivity in the stomach and duodenum to acid, bile acid and certain nutrients may also cause abdominal pain in FD patients [7]. Delayed gastric emptying may cause distal gastric distension, which in turn, causes abdominal pain and fullness [8].

Acupuncture and electroacupuncture (EA), acupuncture with electric stimulation, are used to treat gastrointestinal symptoms and diseases in clinic, such as FD [9–11]. Compared with body acupuncture, auricular acupuncture (penetrate needles into auricle) is much easier to master and less painful; yet equally effective [12]. It has become popular worldwide since Paul Nogier introduced the atlas of auricular acupoints [13]. Previous studies have reported its efficacy in the treatment of FD and management of pain, abdominal bloating and belching [14,15]. However, little is known on mechanisms involved in these ameliorating effects.

In recent years, the relationship between auricular acupuncture and the vagal nerve has been well established [16]. Anatomically, a vagal nerve branch is located on the concha in humans, which may play an important role in the effect of auricular acupuncture [17]. Laboratory rodent research has shown that innervations of the central region of the auricle mainly come from the vagus nerve, while the peripheral region of the auricle is mostly innervated by spinal nerves [18]. Accordingly, auricular acupuncture with proper methods of stimulation in suitable acupoints may directly stimulate the vagal nerve and could evoke responses of visceral organs [19]. In a previous study, auricular electro-acupuncture (AEA) was reported to improve rectal distention-induced gastric dysrhythmias via the vagal pathway in rats [20]. Sympathovagal imbalance, decreased vagal tone and increased sympathetic activity, has been reported in patients with FD [21–23]. It has been postulated that the autonomic dysfunctions could play a role in the development of disturbed gastric motility and perception. Accordingly, we hypothesized that AEA might ameliorate visceral hypersensitivity in a rodent model of FD mediated via a mechanism associated with the regulation of autonomic dysfunctions by the activation of the vagal nerve on the concha.

The aim of this study was therefore to study the effect of AEA at appropriate stimulation parameters on gastric hypersensitivity in a rodent model of FD and to explore possible mechanisms involving autonomic functions.

## Materials and methods

### Animals

Seven-day-old Spraque-Dawley male pups with mother rats were purchased from Charles River Laboratories International, Inc. All rats were housed under conditions of 22°C and 12-hour light/dark cycle (6am-6pm). The mother rats were fed with food and water ad libitum. The study was approved by the Institutional Animal Care and Use Committee at the Veterans Affairs Medical Center, Oklahoma City, Oklahoma.

### FD Model

A previously established rodent model of FD was used in this experiment, described as follows [24]: After a three-day period of acclimation, ten-day-old pups were randomly divided into two groups: one group (FD) was given a gavage of 0.2 ml of 0.1% iodoacetamide (IA, Sigma, US) in 2% sucrose (Sigma,US), while the other group (control) was given only 0.2 ml of 2% sucrose. All pups were sent to their mother rats immediately after gavage and fed normally until they were seven weeks old. During the course, the animals were monitored daily for their overall behaviors, including food intake, activity level, alertness and feces.

### Surgical procedures

At the age of seven weeks, the rats were operated under anesthesia for the placement of an intragastric balloon and recording electrodes.

A balloon was placed into the stomach for gastric distention (GD) using the following procedure. After overnight fasting, the rat was anesthetized with intraperitoneal injection of ketamine (60 mg/kg) and xylazine (7 mg/kg). A median abdominal incision was made and then a balloon (2.5cm in diameter) made from latex condom and fixed to the tip of a long catheter (PE-240) was placed into the gastric fundus without obstructing the pylorus. The catheter was tunneled subcutaneously to exit at the back of the neck.

Then, a pair of stainless steel wires (A&E Medical, Farmingdale, NJ, USA) was placed at the acromiotrapezius muscle for recording the electromyogram (EMG). Another pair of stainless steel wires was placed subcutaneously under the skin of the chest for recording the electrocardiogram (ECG). All connecting wires were tunneled subcutaneously and brought out at the back of the neck.

After the surgical procedure, buprenorphine (0.05 mg/kg) was intramuscularly injected, two times per day for 3 days to provide postoperative pain relief to the animal. The rats were housed in individual cages to protect the catheter and the electrodes wires from being chewed off by other rats. No experiments were performed until the rats were completely recovered from the surgery. Typically one week was found to be sufficient for each animal to completely recover from the surgery.

### Auricular electroacupuncture

For AEA, sterile acupuncture needles (Huatuo, Suzhou, Jiangsu, China) were inserted into the bilateral auricular acupoints which are located between the cymba conchae and cavum conchae [20]. For sham-EA, two needles were inserted at sham-points at the hip of the rat, not at any acupoints or meridians. The needle insertion was performed under 2% Isoflurane (Sigma) inhalation. After rats awoke from anesthesia, they were kept and tested in individual restrainers in which rats could only move their legs slightly but could not turn around. Electrical stimulation was performed via the needles using a digital stimulator (World precision instruments, Sarasota, USA).

### Experimental protocols

Experiments were started 1 week after surgery. Totally, there were 24 rats in this study. Eight control and 10 FD rats were used in Experiment 1 to Experiment 4. Another group of 6 FD rats were tested in Experiment 5. At the end of all experiments, 7 control rats and 13 FD rats were subjected to a gastric emptying test.

**Experiment 1:** *Validation of gastric hypersensitivity in FD rats*. The aim of this experiment was to demonstrate that gastric hypersensitivity was established in FD rats. Eight control rats and 10 FD rats were tested in this experiment. The EMG responses to GD at different pressures (20, 40, 60, and 80 mmHg) were recorded. In addition, behavioral responses based on the abdominal withdrawal reflex (AWR) were observed as described by Al-Chaer *et al*. [25]: 0, no behavioral response to GD; 1, brief head movement followed by immobility; 2, contraction of abdominal muscles; 3, lifting of abdomen; 4, body arching and lifting of pelvic structures.

**Experiment 2:** *Effect of AEA on gastric hypersensitivity*. After 2 days rest, the ten FD rats in Experiment 1 were used for testing the effect of AEA on gastric hypersensitivity. Each rat was studied by applying AEA of two different sets of parameters and sham-EA in three randomized sessions with an interval of 2 days. EMG responses to GD at different pressures and behavioral responses were recorded before and immediately after acute AEA/sham-EA. Two sets of different parameters were used for AEA: Set 1: 0.1s on, 0.4s off, 100Hz, 0.3ms, 0.4–0.5mA; this set of parameters was used in electrical stimulation of the stomach for relieving visceral pain [26]; Set 2: 2s on, 3s off, 25Hz, 0.5ms, 0.4–0.5mA; it was used in electroacupuncture at ST36 and shown to accelerate gastric emptying [27]. Both AEA and sham-EA were performed for 30min. Electrical stimulation was given using parameter set 1 in sham-EA.

**Experiment 3:** *AEA in control rats*. After 2 days rest, the eight control rats in Experiment 1 were also treated by AEA with Parameter #1. EMG responses to GD at different pressures were recorded before and immediately after 30-min AEA.

**Experiment 4:** *Autonomic mechanisms*. After the completion of Experiments 1 to 3, the electrocardiogram (ECG) was recorded for 15 min in the fasting state and 30 min during AEA with Parameter #1 in the same 10 FD used in Experiments 1–3. In the same 8 control rats used in Experiments 1–3, the ECG was recorded for 15 min in the fasting state.

**Experiment 5:** *Naloxone application*. Additional 6 FD rats were used in this experiment. Each rat was studied in two randomized sessions with an interval of 2 days: naloxone only and naloxone+AEA. In the naloxone only session, naloxone (3mg/kg, Sigma, USA) was injected intraperitoneally in the fasting state and 50 min later, the EMG responses to graded GD were recorded. In the naloxone+AEA session, naloxone was administrated intraperitoneally (3mg/kg), 20min later, AEA with Parameter #1 was initiated and 30 min more later, EMG responses to graded GD were recorded with the continuation of AEA. Naloxone is a nonselective opioid receptor antagonist (both central and peripheral) and widely used in animal studies to investigate the involvement of opioid mechanisms. The dosage of naloxone was selected based on a previous electroacupuncture study performed in our lab [28].

**Experiment 6:** *AEA on gastric emptying*. To determine a possible compounding effect of abnormal gastric emptying on gastric sensitivity, a gastric emptying test was performed in 7 of the control rats and 13 of the FD rats at the end of all other experiments.

### Measurements

#### Electromyogram (EMG) recording

The EMG responses to GD at different pressures (20, 40, 60, and 80 mmHg) were recorded using an EMG amplifier (EMG 100C; Biopac systems, Inc, Santa Barbara, CA, USA).The EMG signals were filtered at a cutoff frequency of 300 Hz and digitized with a sampling frequency of 2000 Hz. EMG responses were recorded for 20s without GD, 20s with DG at a pressure of 20mmHg and a resting period of 3 min; this process was repeated until all other pressures (40, 60 and 80mmHg) were tested.

The area under the curve (AUC) of the EMG during each period (baseline, during and after GD) was calculated by the software (Acknowledge; Biopac System, Inc., Santa Barbara CA). The final EMG data were presented as a percent increase against the baseline value.

#### Electrocardiogram(ECG) recording

The ECG was recorded from the chest electrodes via an amplifier (Model 2283 Fti Universal Fetrode Amplifier, UFI, Morro Bay, CA, USA) with a recording range of 1.5Hz to 100 Hz. Heart rate variability (HRV) data were derived from the ECG recording using validated software in our lab [29]. In the ECG recording, R waves were identified and R-R intervals were calculated, then R-R interval data was interpolated at 100 Hz and was finally down-sampled at 8 Hz for spectral analysis. The low frequency band in the power spectrum of the HRV signal (LF: 0.07–0.3 Hz) represents mainly sympathetic activity, while the high frequency band (HF: 0.3–4.0 Hz) represents parasympathetic or vagal activity. The LF/HF ratio is calculated to reflect the sympathovagal balance [29].

### Gastric emptying

After 20h fasting, the rats were given 2g of regular solid food within 10 min. The FD rats were treated with AEA (N = 7) or sham-EA (N = 6) for 30min immediately after food intake and the control rats received no treatment. Ninety min after feeding, the rats were killed by decapitation under an overdose of anesthesia (ketamine 100 mg/kg and xylazine 10 mg/kg, IP) and the gastric content was removed and dried in the air. The gastric emptying was calculated as follows [30]: Gastric emptying (%) = (1-dried weight of food recovered from stomach /weight of food intake) ×100.

### Statistical analysis

All data are presented as mean±SE and were analyzed by SPSS 16.0. Student t-test or 2-way repeated measures ANOVA were used to assess the effects of AEA on various measurements. One way ANOVA with repeated measures was performed to compare the LF/HF ratio among different periods in FD rats. The Wilcoxon test was used to compare ranked data. A P value <0.05 was considered to be statistically significant.

## Results

### Gastric hypersensitivity in FD rats

**Fig 1:**
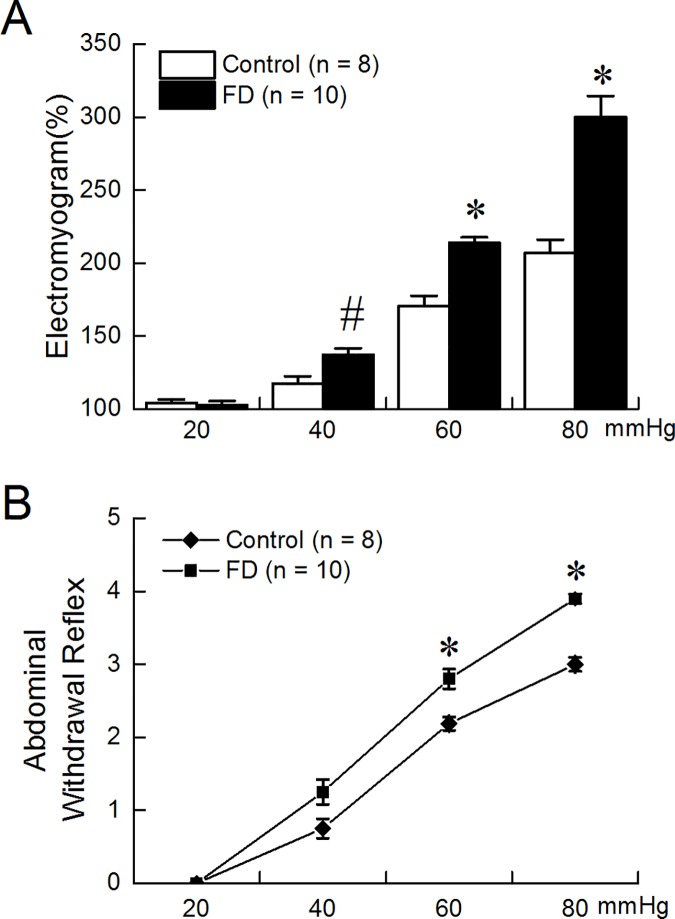
Gastric hypersensitivity in FD rats. EMG responses to GD at 40, 60 and 80mmHg were significantly higher in FD rats than in control rats (*p<0.01, ^#^p<0.05). (B) The behavior responses to GD were significantly higher in FD rats than in control rats (* p<0.01).

EMG responses to GD were significantly higher in the FD rats than in the control rats at distention levels of 40, 60 and 80mmHg (GD at 40, 60 and 80mmHg respectively: 137.08%±4.63% vs.117.39%±4.99%, p = 0.011; 213.85%±3.81% vs. 170.62%±6.99%, p<0.01; 300.06%±14.51% vs. 206.75%±9.34%, p<0.01; Fig 1A). but not at 20mmHg (102.83%±2.56% vs. 104.32%±2.33%, p = 0.68).

The behavior responses to GD were consistent with the EMG data. The FD rats showed higher AWR scores than the control rats when GD was at 60 mmHg (2.80±0.13 vs. 2.19±0.09, p<0.01) and 80mmHg (3.90±0.07 vs. 3.00±0.09, p<0.01) (Fig 1B).

### Ameliorating effect of AEA on gastric hypersensitivity in FD rats

**Fig 2:**
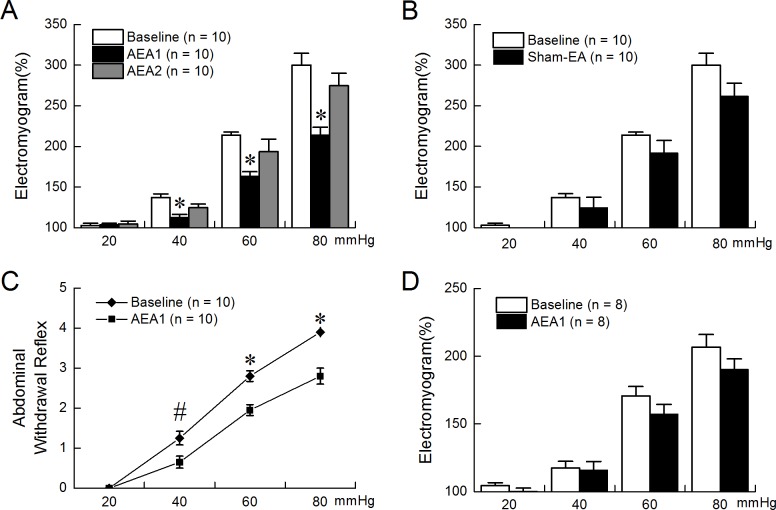
Ameliorating effect of AEA on gastric hypersensitivity. AEA1, but not AEA2, reduced gastric hypersensitivity in FD rats. AEA1 decreased EMG responses to GD at 40, 60 and 80mmHg(AEA1 vs. Baseline, *p<0.01). (B) Sham-EA had no effect on gastric hypersensitivity in FD rats. (C) AEA1 could reduce the behavior responses in FD rats (*p<0.01, ^#^p = 0.018). (D) AEA1 had no effect on gastric sensitivity in control rats.

AEA with Parameter #1 (AEA 1), but not Parameter #2 (AEA 2), reduced gastric hypersensitivity in FD rats (2-way repeated measures ANOVA, AEA 1 vs. Baseline, p<0.01; AEA 2 vs. Baseline, p = 0.146; AEA 1 vs. AEA 2, P = 0.017, Fig 2A). AEA 1 decreased EMG responses to GD at 40, 60 and 80mmHg (AEA 1vs. Baseline, GD at 40, 60 and 80mmHg respectively; 112.37%±4.23% vs. 137.08%±4.63%, p<0.01; 163.08%±5.85% vs. 213.86%±3.81%, p<0.01; 213.93%±9.74% vs. 300.06%±14.51%, p<0.01, Fig 2A), but not at 20mmHg (AEA 1 vs. Baseline, 103.44%±1.97% vs. 102.83%±2.56%, p = 0.99). Sham-EA had no effects on gastric hypersensitivity in the FD rats (2-way repeated measures ANOVA, p = 0.082, Fig 2B).

Similarly, AEA 1 reduced behavioral responses in the FD rats at distention pressures of 40, 60 and 80mmHg (AEA 1 vs. Baseline, GD at 40, 60 and 80mmHg respectively; 0.65±0.15 vs. 1.25±0.17, p = 0.018; 1.95±0.14 vs. 2.80±0.13, p<0.01; 2.80±0.20 vs. 3.90±0.07, p<0.01; Fig 2C).

In the control rats, AEA 1 showed no effects on gastric sensitivity (2-way repeated measures ANOVA, p = 0.109, Fig 2D).

### Autonomic mechanisms of AEA

**Fig 3:**
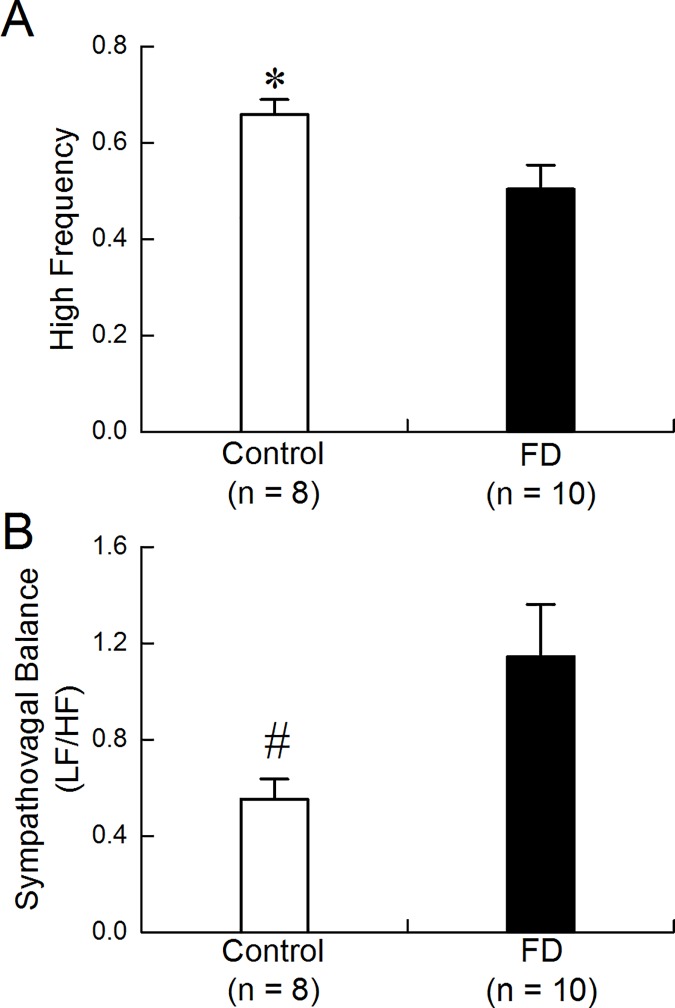
Sympathovagal imbalance in FD rats. The low HF component was found in FD rats (*p = 0.016). (B) The high LF/HF ratio was found in FD rats(^#^p = 0.017).

**Fig 4:**
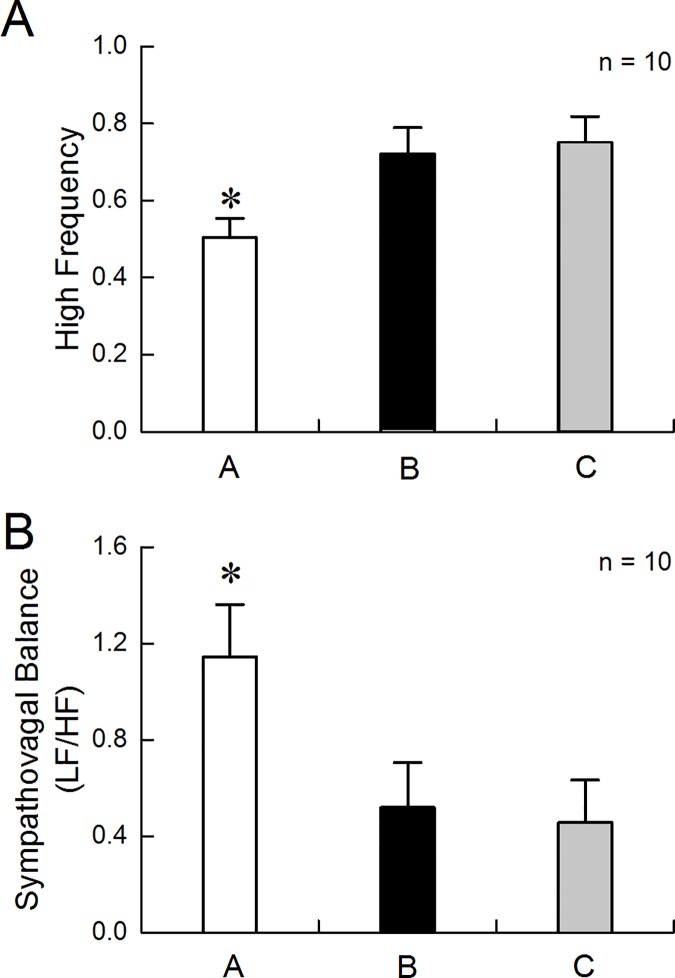
Improvement of sympathovagal balance by AEA1. AEA1 increased the HF component in FD rats (*p<0.01). (B) AEA1 decreased the LF/HF ratio in FD rats (*p<0.01). A in the above figures meant the baseline of HF component or LF/HF ratio. B and C meant the data in the first 15min period and the second 15 min period during AEA1, respectively.

The FD rats exhibited decreased vagal activity and increased sympathovagal balance; these were improved with AEA 1. Spectral analysis of the HRV revealed a lower HF component and higher LF/HF ratio in the FD rats, compared with the control rats (HF component: 0.50±0.05 vs. 0.66±0.03, p = 0.016; Fig 3A. LF/HF ratio: 1.14±0.22 vs. 0.55±0.08, p = 0.017; Fig 3B). However, the HF component was increased after AEA1 in the FD rats (one way ANOVA with repeated measure, A vs. B: 0.50±0.05 vs. 0.72±0.07, p = 0.017; A vs. C: 0.50±0.05 vs. 0.75±0.07, p<0.01, Fig 4A).

Furthermore, the LF/HF ratio was decreased after AEA1 (one way ANOVA with repeated measure, A vs. B: 1.14±0.22 vs. 0.52±0.19, p = 0.047; A vs. C: 1.14±0.22 vs. 0.46±0.18, p<0.01, Fig 4B). There were no statistical differences between the first 15min period and the second 15min in the HF component and LF/HF ratio during AEA1.

### Naloxone did not block the effect of AEA

**Fig 5:**
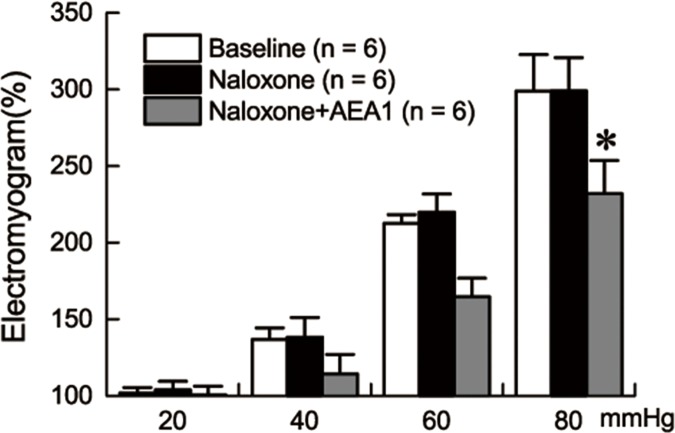
Naloxone did not block the effect of AEA1. Naloxone did not influence the EMG responses to GD. AEA1 decreased the EMG responses when naloxone was performed intraperitoneally 20min before AEA1(*p = 0.01).

Naloxone did not block the ameliorating effect of AEA on gastric hypersensitivity to GD. Naloxone alone did not influence the EMG response to GD in the FD rats (2-way repeated measures ANOVA, p = 0.726, Fig 5). AEA1 was still able to decrease the EMG responses to graded GD in the FD rats at the presence of naloxone (2-way repeated measures ANOVA, naloxone+AEA1 vs. naloxone, p = 0.010, Fig 5).

### AEA did not alter gastric emptying

**Fig 6:**
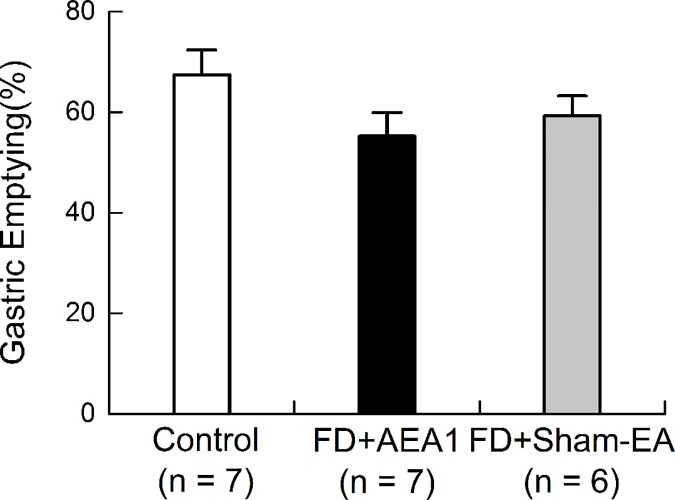
AEA1 did not alter gastric emptying. There was no significant difference in gastric emptying among control group, FD+ AEA1 group and FD+sham-EA group.

Gastric emptying was found to be same in the control and FD rats, and was not affected by AEA. There was no statistical difference in gastric emptying between the FD rats with sham-EA and control rats (FD with sham-EA vs. Control: 59.26%±3.96% vs. 67.41%±4.94%, p = 0.453, Fig 6). AEA did not change gastric emptying in the FD rats, compared with sham-EA (AEA vs. sham-EA: 55.25%±4.69% vs. 59.26%±3.96%, p = 0.820, Fig 6).

## Discussion

In this study, we found that AEA with proper parameters decreased gastric hypersensitivity in FD rats without altering gastric emptying. This ameliorating effect of AEA might be mediated via the regulation of abnormal autonomic functions in FD rats. Naloxone did not block the ameliorating effect of AEA on gastric hypersensitivity in FD rats, excluding the involvement of the opioid pathway.

EA has been widely accepted as an alternative method for the treatment of pain, such as migraine [31], neuropathic pain [32], visceral pain [33], and inflammatory pain [34]. However, AEA is another auxiliary acupuncture method that inserts needle into the auricle. Important progress has been made in recent years in both basic and clinical research on auricular acupuncture or AEA. Several diseases or symptoms can be treated or relieved by auricular acupuncture or AEA alone [35–37]. It is speculated that auricular acupuncture may become one of the most promising acupuncture therapies since it is much easier to apply, less painful, safer and equally effective [12], compared with body acupuncture.

The rodent model of FD used in this study was based on a high quality published paper [24]: gastric hypersensitivity is established via neonatal gastric irritation using IA. It was originally reported that when the rats reached to adulthood, they exhibited higher gastric sensitivity to GD. This was also observed in this study: the FD rats expressed gastric hypersensitivity when GD was performed at 40, 60 and 80mmHg. AEA with Parameter #1 decreased gastric hypersensitivity in the FD rats but not in the control rats. According to the theory of Chinese Traditional Medicine, AEA is believed to be capable of restoring the balance of Yin and Yang [38] and improving the pathological state, but cannot change the normal stable state.

An important component of this study was to optimize stimulation parameters. It was observed that Parameter #1, but not Parameter #2, decreased gastric hypersensitivity. The importance of using optimal parameters of EA has been proven by Han *et al* [39]. They have shown that a combination of two frequencies (2Hz and 100Hz) produce a simultaneous release of all four opioid peptides, enkephalin, beta-endorphin, endomorphin and dynorphin. The release of these opioids resulted in the maximal therapeutic effect in patients with various kinds of chronic pain, including low back pain and diabetic neuropathic pain. Based on their findings, we designed a new set of parameters (intermittent pulse trains with train on time of 0.1s and off time of 0.4s, and a pulse frequency of 100Hz in each pulse train); this provided a train frequency of 2Hz and a pulse frequency of 100Hz. Sun *et al* [26] have shown that gastric electrical stimulation with this set of parameters reduced visceral hypersensitivity in rats with gastric ulcers. The effectiveness of this set of parameters was further supported by the current study. The other set of parameters (2s on, 3s off, 25Hz) has been commonly used in EA and consistently shown to improve gastrointestinal motility, such as delayed gastric emptying [27]. However, it was shown to be ineffective in treating gastric hypersensitivity in the present study. These results demonstrated the specificity of AEA and the importance of parameter optimization in clinical applications.

Delayed gastric emptying may cause gastric distension, which in turn, induces gastric pain. In order to exclude this possible compounding effect on gastric sensitivity, gastric emptying in both control and FD rats was measured at the end of the experiments. It was found that gastric emptying was not altered with either neonatal insult (no difference between control and FD rats) with IA or AEA (no difference between AEA1 and sham EA). Thus, the observed ameliorating effect of AEA on gastric hypersensitivity was not attributed to alternations in gastric emptying.

The autonomic mechanism was discovered to contribute to the ameliorating effect of AEA on gastric hypersensitivity in this study. The abnormality of autonomic nervous function may play a role in the pathogenesis of FD. A lower vagal tone and higher sympathetic tone were reported in FD patients, compared with healthy persons [21–23]. EA has been consistently shown to regulate autonomic function in clinical and animal studies [27, 40]. Previously AEA on the concha of the ear was shown to increase vagal activity in healthy men [41]. In another clinical research, auricular acupuncture reduced the LF/HF (sympathetic/vagal) ratio and increased HF (vagal) during the postoperative period in patients who had undergone hemicolectomy [42]. In the present study, the HF component was much lower and the LF/HF ratio was higher in the FD rats compared with the controls, echoing the previous clinical findings. As expected, AEA increased the HF component and decreased the LF/HF ratio.

Our findings suggested a vagal afferent mechanism; however, the exact afferent pathway was not studied in the present study. The enhancement of cardiac vagal efferent activity seemed to suggest the activation of neurons in the nucleus ambiguous and the dorsal motor nucleus of the vagus via the auricular vagal afferent and the nucleus tractus solitarii pathway. Since gastric emptying was not altered, the neurocircuit between the ear and the dorsal motor nucleus might involve a discrete subset of motorneurons that are not involved in gastric emptying. These speculations need to be elucidated in future studies.

The opioid pathway is one of important mechanisms involved in the analgesic effect of acupuncture or EA [43, 44]; however, the results of the present study did not support this observation. In addition, the opioids have also been reported to play a role in the modulation of sympathetic activity [45, 46]. The sympathetic nervous system was reported to be inhibited both postsynaptically and presynaptically by opioid peptides produced in the heart via their respective receptors [45]. In another study, chronic μ-opioid receptor stimulation by methadone was noted to decrease resting efferent sympathetic nerve activity to muscle [46]. It is generally accepted that activation of the opioid pathway suppresses sympathetic outflow from the brain to the periphery. In a previous study in our lab, the prokinetic effect of electroacupuncture at ST36 was found to be mediated via both the autonomic and opioid pathways. Accordingly, one experiment in this present study was designed to elucidate whether the opioid mechanism was also involved in the ameliorating effect of AEA on gastric hypersensitivity. However, the findings of this study seemed to indicate that the improvement in autonomic function with AEA was independent of the opioid pathway. This could be attributed to the direct innervation of the autonomic nerves in the ear.

Although gastric pain is common in FD, treatment options are limited. The findings of this present study suggested a therapy potential of AEA for treating gastric pain in patients with FD. Follow-up clinical studies to explore this potential are warranted. Moreover, with appropriate methodologies/stimulation parameters auricular acupuncture and AEA may also be applicable to treat a more general problem of visceral pain [47].

In conclusion, AEA has an ameliorating effect on gastric hypersensitivity in a rodent model of FD and this effect may be attributed to the improvement of sympathovagal balance.
